# Pathogenic sequence variant and microdeletion affecting *HMGA2* in Silver–Russell syndrome: case reports and literature review

**DOI:** 10.1186/s13148-024-01688-w

**Published:** 2024-06-05

**Authors:** Kaori Yamoto, Hirotomo Saitsu, Yumiko Ohkubo, Masayo Kagami, Tsutomu Ogata

**Affiliations:** 1 Department of Biochemistry, Hamamatsu University School of Medicine, 1-20-1, Handayama, Chuo-ku, Hamamatsu, 431-3192 Japan; 2 Department of Pediatrics, Hamamatsu University School of Medicine, Hamamatsu, Japan; 3 Department of Pediatrics, Shizuoka Saiseikai Hospital, Shizuoka, Japan; 4 Department of Molecular Endocrinology, National Research Institute for Child Health and Development, Tokyo, Japan; 5 Department of Pediatrics, Hamamatsu Medical Center, Hamamatsu, Japan

**Keywords:** *HMGA2*, Silver–Russell syndrome, Whole exome sequencing, 12q14 microdeletion, *IGF2*

## Abstract

Silver–Russell syndrome (SRS) is a representative imprinting disorder characterized by pre- and postnatal growth failure. We encountered two Japanese SRS cases with a de novo pathogenic frameshift variant of *HMGA2* (NM_003483.6:c.138_141delinsCT, p.(Lys46Asnfs*16)) and a de novo ~ 3.4 Mb microdeletion at 12q14.2–q15 involving *HMGA2*, respectively. Furthermore, we compared clinical features in previously reported patients with various genetic conditions leading to compromised *IGF2* expression, *i.e.*, *HMGA2* aberrations, *PLAG1* aberrations, *IGF2* aberrations, and *H19*/*IGF2*:IG-DMR epimutations (hypomethylations). The results provide further support for *HMGA2* being involved in the development of SRS and imply some characteristic features in patients with *HMGA2* aberrations.

**Supplementary Information:**

The online version contains supplementary material available at 10.1186/s13148-024-01688-w.

## Introduction

Silver–Russell syndrome (SRS; MIM #180860) is a representative imprinting disorder characterized by pre- and postnatal growth failure [1]. SRS is clinically diagnosed when a patient is positive for ≥ four of six items (low birth weight and/or length, postnatal growth failure, relative macrocephaly, prominent forehead, body asymmetry, and feeding difficulties and/or low body mass index) utilized in the Netchine-Harbison clinical scoring system (N–H CSS) (Table 1) [1]. SRS is a genetically heterogeneous condition, with the most common genetic defect being epimutation (hypomethylation) of the paternally inherited *H19/IGF2*:IG-differentially methylated region (DMR) and resultantly compromised *IGF2* expression identified in 30–60% of patients, followed by maternal uniparental disomy for chromosome 7 (UPD(7)mat) observed in 5–10% of patients [1]. In addition, other genetic abnormalities including UPD(16)mat and UPD(20)mat and sequence variants in multiple genes such as *IGF2*, *CDKN1C*, *PLAG1*, and *HMGA2*, have also been revealed in SRS [1, 2].

**Table 1:** Clinical findings in patients with various SRS-related underlying factors

	Case 1	Case 2	Group 1	Group 2	Group 3	Group 4	Group 5	Group 6	*P*-value		
			*HMGA2* intragenic sequence variants/deletions (n = 24)	*HMGA2*-containing deletions (n = 23)	*PLAG1* intragenic sequence variant (n = 11)	*PLAG1*-containing deletions (n = 5)	*IGF2* intragenic sequence variant (n = 14)	*H19/IGF2*:IG-DMR epimutations (n = 43 ~ 226)	Group 1 *vs.* Group 3	Group 1 *vs.* Group 5	Group 1 *vs.* Group 6
*Netchine-Harbison scoring system features for Silver–Russell syndrome*											
Diagnosis of SRS (≥ 4/6)	+	+	13/23 (56.5%)	7/22 (31.8%)	5/11 (45.5%)	2/5 (40%)	14/14 (100%)	43/43 (100%)	0.72	**0.0056**	**0.0000054**
Birth length and/or weight $\le$ − 2 SDS	+	+	19/21 (90.5%)	15/21 (71.4%)	11/11 (100%)*	3/5 (60%)*	14/14 (100%)	35/35 (100%)	0.53	0.51	0.14
Postnatal height $\le$ − 2 SDS (~ 2 years)	+	+	23/23 (100%)	22/22 (100%)	7/7 (100%)	2/4 (50%)	14/14 (100%)	145/173 (83.8%)	1.00	1.00	0.0503
Relative macrocephaly at birth	+	+	6/16 (37.5%)	5/22 (22.7%)	4/7 (57.1%)	3/5 (60%)	14/14 (100%)	111/112 (99.1%)	0.65	**0.00030**	**0.0000037**
Prominent forehead (1–3 years)	+	–	15/20 (75%)*	6/22 (27.3%)*	6/7 (85.7%)	4/5 (80%)	14/14 (100%)	118/126 (93.7%)	1.00	0.063	0.018
Body asymmetry	–	–	2/18 (11.1%)	0/22 (0%)	0/9 (0%)	0/5 (0%)	3/14 (21.4%)^†^	175/226 (77.4%)^†^	0.54	0.63	**0.00**
Feeding difficulties and/or low BMI	+	+	16/19 (84.2%)	15/16 (93.8%)	9/10 (90%)	4/4 (100%)	14/14 (100%)	124/173 (71.7%)	1.00	0.24	0.29
*Pregnancy and delivery*											
Gestational age (weeks)	39	39	39 (29 ~ 42) (n = 18)	40 (27 ~ 42) (n = 20)	39 (31 ~ 40) (n = 11)	36 (30 ~ 38) (n = 5)	36 (29 ~ 43) (n = 14)	38 (34 ~ 40) (n = 36)	0.74	0.068	…
Placental weight (SDS)	− 0.82	− 1.65	…	…	…	…	− 2.7 ± 0.5 (n = 4)	− 2.1 ± 0.7 (n = 14)	…	…	…
Hypoplastic placenta (< 80%)	–	+	…	…	…	…	4/4 (100%)	11/12 (91.6%)	…	…	…
Oligohydramnios	+	–	…	…	…	…	3/6 (50.0%)	7/11 (63.6%)	…	…	…
*Craniofacial features not involved in Netchine-Harbison criteria*											
Triangular face	+	+	13/18 (72.2%)	9/22 (40.9%)	8/9 (88.9%)	5/5 (100%)	14/14 (100%)	73/74 (98.6%)	0.62	0.052	**0.00092**
Low set ears or ear anomalies	–	–	3/17 (17.6%)	3/21 (14.3%)	0/5 (0%)	3/5 (60%)	7/11 (63.6%)	70/140 (50.0%)	1.00	0.02	0.018
Cleft palate	–	–	0/15 (0%)	1/21 (4.8%)	0/5 (0%)	0/5 (0%)	6/14 (42.8%)	…	1.00	**0.0063**	…
Micrognathia	+	+	2/15 (13.3%)	8/21 (38.1%)	1/5 (20%)	1/5 (20%)	8/8 (100%)	59/79 (74.7%)	1.00	**0.000091**	**0.000012**
*Limb/digital features*											
Long bone deficiency	–	–	0/18 (0%)	0/21 (0%)	0/5 (0%)	0/5 (0%)	1/14 (7.1%)	… (0%)	1.00	0.44	…
Ectrodactyly	–	–	0/18 (0%)	0/21 (0%)	0/5 (0%)	0/5 (0%)	3/14 (21.4%)	… (0%)	1.00	0.073	…
Polydactyly	–	–	0/18 (0%)	0/21 (0%)	0/5 (0%)	0/5 (0%)	1/14 (7.1%)	… (0%)	1.00	0.44	…
Syndactyly	–	–	2/18 (11.1%)	0/21 (0%)	0/5 (0%)	0/5 (0%)	5/14 (35.7%)	59/141 (41.8%)	1.00	0.19	0.0106
Clinodactyly	+	+	6/18 (33.3%)	5/21 (23.8%)	1/5 (20%)	3/5 (60%)	12/14 (85.7%)	142/176 (80.7%)	1.00	**0.0045**	**0.000055**
Osteopoikilosis	…	+	…	7/20 (35.0%)	…	…	…	…	…	…	…
Supplemental references: Additional file 7 [This may be necessary]			1–13	14–25	26–31	32–35					

*P*-values < 0.05 are boldfaced

BMI, body mass index

For the frequency, the denominators indicate the number of patients examined for the presence or absence of each feature, and the numerators represent the number of patient assessed to be positive for that feature

Statistical significance of the frequency was examined by the Fisher’s exact probability test, and that of the median by the Mann–Whitney’s *U* test

Clinical description remain quite fragmentary in a single case in group 1 and in a single case in group 2

Two cases in group 1 are homozygotes for *HMGA2* missense variants

Detailed phenotypes in groups 1–4 are shown in Supplemental Tables 2–5, respectively

Data in groups 5 and 6 are derived from Masunaga et al. 2020

^*^P < 0.05 for prominent forhead between groups 1 and 2 and for birth lenghth and/or weight –2 SDS between groups 3 and 4

^†^*P* < 0.001 for body asymmetry between groups 5 and 6

*HMGA2* (High Mobility Group AT-hook 2; MIM *600,698) on chromosome 12q14.3 is a transcriptional factor gene that plays a critical role in fetal growth and development [2]. HMGA2 exaggerates not only *IGF2* expression but also *PLAG1* expression, and PLAG1 enhances *IGF2* expression [2]. Thus, *HMGA2* functions as a direct and indirect (PLAG1-mediated) positive regulator for *IGF2* which constitutes a major causative gene for SRS. Consistent with this, intragenic loss-of-function sequence variants and microdeletions of *HMGA2* are frequently associated with SRS-compatible phenotype [2], as are 12q14 microdeletions involving *HMGA2* [3]. However, detailed clinical features remain to be clarified in patients with pathogenic sequence variants and microdeletions affecting *HMGA2*.

Here, we report two hitherto undescribed SRS cases with a pathogenic sequence variant and a microdeletion affecting *HMGA2*, respectively, and compare clinical features in previously reported patients with various genetic conditions leading to compromised *IGF2* expression. The results imply some characteristic features in patients with *HMGA2* abnormalities.

## Case presentation

### Case 1

**Fig. 1:**
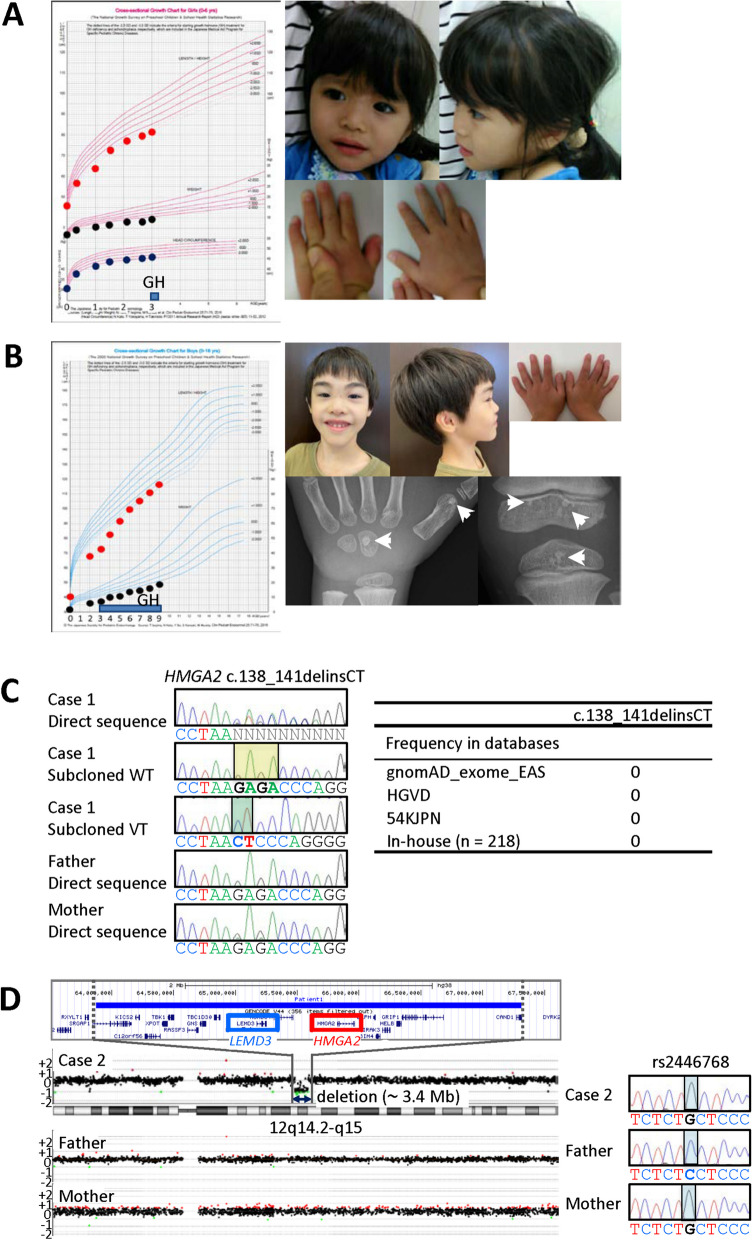
Clinical and molecular findings of cases 1 and 2. **A** Growth pattern and photographs of case 1 at two years of age. Growth hormone therapy has been started at three years of age. **B** Growth pattern and photographic and roentgenographic images of case 2 at seven years of age. Growth hormone therapy has been started (blue box) at three years of age. Osteopoikilosis is indicated by arrows. **C** The pathogenic sequence variant identified in case 1. Left panel: Sanger sequencing of case 1 and her parents, and the subcloned wildtype (WT) and variant (VT) sequences of case 1. A de novo heterozygous indel variant (c.138_141delinsCT) in *HMGA2* is shown in case 1. Deleted and inserted nucleotides are highlighted with yellow and green rectangles, respectively. Right panel: Frequencies in the databases. **D** The pathogenic microdeletion identified in case 2. Left panel: Genomewide aCGH analysis showing a heterozygous ~ 3.4 Mb microdeletion at 12q14.2–q15 (displayed by UCSC genome browser (http://genome.ucsc.edu)). His parents have no microdeletion. Right panel: Sanger sequencing of rs2446768 (chr12:65,845,937 on the deleted region) (highlighted with light blue rectangle). This shown that the microdeletion has occurred on the paternally inherited chromosome

Growth pattern and photographs are shown in Fig. 1A. Case 1 was a Japanese girl born to unrelated parents at 39 weeks of gestation. The pregnant course was complicated by oligohydramnios, although placental weight of 432 g was within the normal range (82% of the mean placental weight of 527 g at 37–40 weeks of gestation [4]). Her parents were healthy and normal in height (the father 171 cm, ± 0.0 SD; the mother 157 cm, − 0.2 SD).

At birth, her length was 46.0 cm (− 1.7 SD), her weight 2140 g (− 2.9 SD), and her occipitofrontal circumference (OFC) 31.5 cm (− 1.4 SD). She exhibited triangular face, prominent forehead, almond-shaped palpebral fissure, concave nasal ridge, micrognathia, small hands, and fifth finger clinodactyly, but lacked body asymmetry. She showed postnatal growth failure with a height of 75.0 cm (− 3.2 SD) and a weight of 7.4 kg (− 4.2 SD) at two years of age. She had feeding difficulty during infancy. Thus, case 1 was positive for five of six N–H CSS items, except for body asymmetry (Table 1). Her mental development appeared normal. At 3 0/12 years of age, her height was 81.6 cm (− 3.2 SD), her weight 9.3 kg (− 3.4 SD), and her OFC 46.0 cm (− 1.6 SD); thus, she satisfied the Japanese criteria to receive GH therapy for patients who were born small for gestational age (SGA) and remained short at three years of age. At present, she is 3 3/12 years old, and has been placed on GH therapy with a dosage of 0.23 mg/kg/week.

### Case 2

Growth pattern and photographic and roentgenographic images are shown in Fig. 1B. Case 2 was a Japanese boy born to non-consanguineous parents as the third child at 39 weeks of gestation, after an uncomplicated pregnancy and delivery. Placenta weighed 348 g (66% of the gestational age-matched mean placental weight [4]). The parents were clinically unremarkable with normal heights (the father 172 cm, + 0.2 SD; the mother 162 cm, + 0.8 SD).

At birth, his length was 40.0 cm (− 4.2 SD), his weight 2.0 kg (− 3.4 SD), and his OFC 32.0 cm (− 0.9 SD). Physical examination revealed triangular face, micrognathia, small hands, and fifth finger clinodactyly, but not prominent forehead and body asymmetry. He showed persistent postnatal growth failure, with a height of 67.7 cm (− 5.7 SD) and a weight of 6.15 kg (− 6.9 SD) at two years of age. He exhibited feeding difficulty during infancy. Thus, he was positive for four of the six N–H CSS items, except for prominent forehead and body asymmetry (Table 1). His developmental milestones were grossly normal: he walked without support at 18 months of age and spoke two-word sentences at 30 months of age. GH therapy for SGA short stature was started from 3 years of age, with the GH dosage being gradually increased from 0.23 to 0.30 mg/kg/week, improving statural growth. After revealing a microdeletion involving *HGMA2* and *LEMD3*, bone survey was performed at seven years of age, showing osteopoikilosis characterized by circular osteosclerotic dysplasia. At present, he is nine years and seven months old, and shows upward growth shift with a height of 120.6 cm (− 2.3 SD), a weight of 20.7 kg (− 2.6 SD), and an OFC of 51.8 cm (− 0.9 SD).

### Molecular studies

We performed comprehensive molecular studied for SRS including methylation analyses of multiple SRS-related DMRs, genomewide copy number variation (CNV) analysis, and whole exome sequencing (WES), as reported by Inoue et al. [5], using leukocyte genomic DNA samples from cases 1 and 2 and their parents. Methylation analyses was carried out using pyrosequencing or methylation-specific multiplex ligation-dependent probe amplification. Genomewide CNV analysis was performed by array comparative genomic hybridization (aCGH) using a 60 K catalog array (SurePrint G3 Human CGH) (Agilent Technologies). WES was carried out using the SureSelectXT Human All Exon v6 (Agilent Technologies), and captured library was sequenced on NextSeq500 (Illumina) with 150 bp paired-end reads. Exome data processing, variant calling, annotation, and filtering were performed, as reported previously [6], using Human GRCh38/hg38 as the reference genome.

Methylation analyses revealed no abnormal methylation pattern in SRS-related DMRs of cases 1 and 2. Subsequently, case 1 was found to have a de novo indel variant leading to frameshift and premature termination in exon 2 of *HMGA2* (NM_003483.6:c.138_141delinsCT, p.(Lys46Asnfs*16)) which was confirmed by Sanger direct sequencing and sequencing of the subcloned wildtype and variant alleles (Fig. 1C). This variant was absent from the public and in-house databases utilized in this study, and satisfied the condition to undergo nonsense-mediated mRNA decay (NMD) [7]. The paternity and maternity were confirmed by PLINK analysis (Additional file 2: Table S1). Case 2 was revealed to have a de novo ~ 3.4 Mb microdeletion at 12q14.2–q15 (chr12:63,871,180–67,314,583) by aCGH analysis (Fig. 1D). This microdeletion involved 18 protein-coding genes including *HMGA2* and *LEMD3*, and was demonstrated to have occurred in chromosome 12 of paternal origin by genotyping of SNPs on the deleted region (Fig. 1D). No other abnormal molecular finding related to SRS was identified in cases 1 and 2.

## Discussion

We revealed a frameshift variant in *HMGA2* and a ~ 3.4 Mb microdeletion involving *HMGA2* in cases 1 and 2, respectively, with clinically diagnosed SRS. According to the ACMG/AMP guideline, the frameshift variant is evaluated as pathogenic, because it is positive for PVS1 (null variant), PS2 (de novo occurrence with confirmed paternity and maternity), and PM2_supporting (absence in the control populations) [8]. In addition, the frameshift variant is predicted to undergo NMD [7]. Similarly, the de novo microdeletion involving *HMGA2* is considered to be a deleterious CNV leading to the development of SRS. The results provide further support for *HMGA2* being the causative gene for SRS.

To our knowledge, 21 different *HMGA2* intragenic sequence variants/microdeletions (four missense, eight nonsense/frameshift, and four intronic sequence variants, and five microdeletions involving an exon(s)) have been identified in 24 patients (group 1) (Additional file 1: Fig. S1), and 18 different *HMGA2*-containing microdeletions ranging from 1.35 to 10.12 Mb in size have been revealed in 23 patients (group 2), including cases 1 and 2 in this study. In this regard, two findings are notable. First, while most sequence variants are present in a heterozygous condition, a missense variant (c.239C > T, p(Pro80Leu)) is present in a homozygous condition in two siblings with SRS features accompanied by severe short stature (~ − 6 SD) and in a heterozygous condition in their parents with borderline short stature [9]. This implies a gene dosage effect of *HMGA2* intragenic sequence variants in the development of SRS phenotype. Second, the microdeletion in case 2 has occurred in the paternally inherited chromosome 12. This would argue against the possible relevance of some imprinting mechanism in the phenotypic development implicated by the finding that seven familial sequence variants and three familial microdeletions affecting *HMGA2* reported to date are invariably of maternal origin [3] (Additional files 3 and 4: Table S2 and Table S3).

Clinical features of groups 1 and 2 are summarized in Table 1, and detailed findings of each patient are shown in Additional files 3 and 4: Table S2 and Table S3. Two findings are notable in the phenotypic comparison between groups 1 and 2. First, SRS-like clinical features including triangular face tend to be more frequent in group 1 than in group 2, with a significant difference for prominent forehead, whereas other features such as cleft palate and micrognathia tend to be more frequent in group 2 than in group 1. This would imply that deletion or disruption of multiple genes other than *HMGA2* have obscured SRS phenotype and facilitated non-SRS phenotype in group 2. This would also explain why intellectual disability has frequently been observed in group 2 [3]. Second, osteopoikilosis is frequently and exclusively found in group 2. In this regard, *LEMD3* (MIM *607,844) is known as a causative gene for osteopoikilosis, because pathogenic loss-of-function variants in *LEMD3* have been demonstrated in several families with autosomal-dominant osteopoikilosis [10]. Indeed, osteopoikilosis in group 2 has been identified only in patients missing *LEMD3*, with a reduced penetrance [3].

To reveal clinical characteristics in *HMGA2*-related SRS, we first compared clinical findings between *HMGA2*-related SRS and *PLAG1*-related SRS which is divided into *PLAG1* intragenic sequence variants (group 3) and *PLAG1*-containing microdeletions (group 4) (detailed findings of each patient are shown in Additional files 5 and 6: Table S4 and Table S5, respectively). Clinical findings are grossly comparable between groups 3 and 4, except for the frequency of low birth weight and/or length, although the number of patients remains small especially in group 4. Notably, clinical features are quite similar between group 1 and group 3 with no significant difference (Table 1). This would imply that *HMGA2* intragenic sequence variants/microdeletions reduce *IGF2* expression primarily by compromising a PLAG1-mediated indirect regulatory function, rather than a direct regulatory function, for *IGF2* expression. In this regard, previous studies have suggested that *IGF2* expression is more severely affected in *HMGA2* sequence variants than in *PLAG1* sequence variants and, consistent with this, patients with *HMGA2* sequence variants appear to show more typical SRS body parameters than those with *PLAG1* sequence variants [2]. Thus, it is likely that the degree of SRS phenotype is more severe in patients with *HMGA2* sequence variants than in those with *PLAG1* sequence variants, but the frequency of SRS phenotype is similar between the two groups of patients.

We next compared clinical findings of group 1 with those of previously reported *IGF2* intragenic sequence variants (group 5) and *H19*/*IGF2*:IG-DMR epimutations (group 6) (Table 1) (for comparison between groups 5 and 6, see Masunaga et al. [6]). Notably, the frequency of patients satisfying the N–H CSS and that of patients with relative macrocephaly at birth is far higher in groups 5 and 6 than in group 1, as is that of patients with other skeletal features. This would imply that *IGF2* expression is more severely compromised in groups 5 and 6 than in group 1 (as well as in group 3), as reported previously [2]. In addition, body asymmetry (hemihypoplsia) is highly prevalent in group 6. This is consistent with the mosaicism consisting of cells with normally methylated *H19*/*IGF2*:IG-DMR and those with hypomethylated *H19*/*IGF2*:IG-DMR in group 6, because epimutation (hypomethylation) is primarily caused by defective methylation maintenance in the postzygotic mitosis [1].

In summary, the present study suggests that *HMGA2* aberrations lead to SRS phenotype with a similar frequency to *PLAG1* aberrations, and less frequently than *IGF2* intragenic sequence variants and *H19*/*IGF2*:IG-DMR epimutations. Further studies will permit a better clarification of molecular and clinical characters in *HMGA2* aberrations.

### Supplementary Information

**Additional file1: Fig. S1**. HMGA2 intragenic sequence variants and microdeletions reported to date. HMGA2 consists of five exons, and coding and noncoding regions are shown with black and white boxes, respectively. **Additional file 2. Table S1**. Assessment of paternity and maternity based on PI_HAT values calculated by PLINK. **Additional files 3–6. Tables S2–S5**. Clinical features of patients with HMGA2 intragenic sequence variants/microdeletions (group 1) (Table S2) and HMGA2-containing microdeletions (group 2) (Table S3), and those of previously reported patients with PLAG1 sequence variants (group 3) (Table S4) and PLAG1-containing microdeletions (group 4) (Table S5). **Additional file 7**. Supplementary references for Tables S2–S5.

## Data Availability

The study data that support our findings will be made available to qualified investigators upon reasonable request.

## Abbreviations

aCGH: Array comparative genomic hybridization
CNV: Copy number variation
DMR: Differentially methylated region
*HMGA2*: High Mobility Group AT-hook 2
N–H CSS: Netchine–Harbison clinical scoring system
SGA: Small for gestational age
SRS: Silver–Russell syndrome
UPD: Uniparental disomy
WES: Whole exome sequencing
