# Supplementary material for: Pathogenic sequence variant and microdeletion affecting HMGA2 in Silver–Russell syndrome: case reports and literature review
Source: Clin Epigenetics. 2024 Jun 5;16:73. doi: 10.1186/s13148-024-01688-w (PMC11155105; doi:10.1186/s13148-024-01688-w)
Supplement: Supplementary file 1 — Additional file1: Fig. S1. HMGA2 intragenic sequence variants and microdeletions reported to date. HMGA2 consists of five exons, and coding and noncoding regions are shown with black and white boxes, respectively. Additional file 2. Table S1. Assessment of paternity and maternity based on PI_HAT values calculated by PLINK. Additional files 3–6. Tables S2–S5. Clinical features of patients with HMGA2 intragenic sequence variants/microdeletions (group 1) (Table S2) and HMGA2-containing microdeletions (group 2) (Table S3), and those of previously reported patients with PLAG1 sequence variants (group 3) (Table S4) and PLAG1-containing microdeletions (group 4) (Table S5). Additional file 7. Supplementary references for Tables S2–S5. [file 13148_2024_1688_MOESM1_ESM.pdf]

Supplementary Materials

Additional file 1. Supplementary Figure S1. *HMGA2* intragenic sequence variants and microdeletions reported to date. *HMGA2* consists of five exons, and coding and non-coding regions are shown with black and white boxes, respectively.

Additional file 2. Supplementary Table S1. Assessment of paternity and maternity based on PI_HAT values calculated by PLINK.

Additional files 3–6. Supplementary Tables S2–S5. Clinical features of patients with *HMGA2* intragenic sequence variants/microdeletions (group 1) (Table S2) and *HMGA2*-containing microdeletions (group 2) (Table S3), and those of previously reported patients with *PLAG1* sequence variants (group 3) (Table S4) and *PLAG1*-containing microdeletions (group 4) (Table S5).

Additional file 7. Supplementary references for Tables S2–S5.

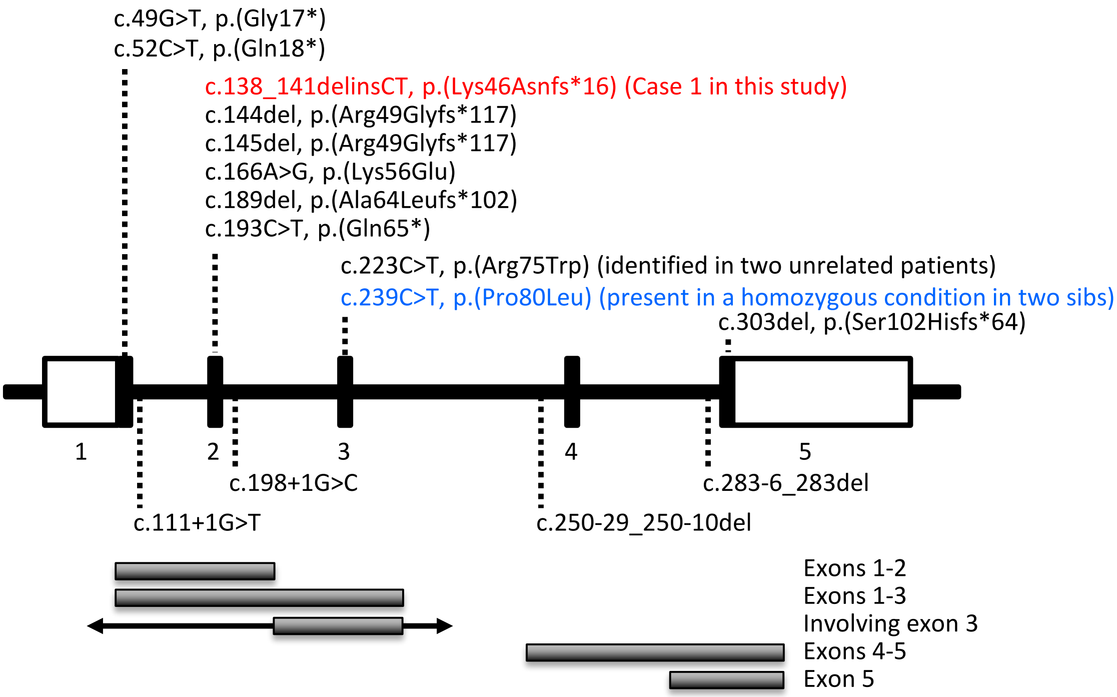

Supplementary Figure S1. *HMGA2* intragenic sequence variants and microdeletions reported to date. *HMGA2* consists of five exons, and coding and non-coding regions are shown with black and white boxes, respectively.

Supplementary Table S1. Assessment of paternity and maternity based on PI_HAT values calculated by PLINK

IID1	IID2	Z0	Z1	Z2	PI_HAT
Patient	Father	0	1	0	0.5
Patient	Mother	0	1	0	0.5
Father	Mother	1	0	0	0

- A total of 6,908 common exonic variants of whole exome sequencing data of the patient and the parents and those of 359 individuals from 1000 genome project have been jointly analyzed by PLINK 1.9 beta (https://www.cog-genomics.org/plink/1.9/).
- IID1: Individual ID for first individual; IID2: Individual ID for second individual; Z0: P(IBD=0) (IBD: identity by descent); Z1: P(IBD=1); Z2: P(IBD=2); and PI_HAT: P(IBD=2)+0.5*P(IBD=1) (proportion IBD). IBD: identical by descent.
- Theoretical PI_HAT value is 1.0 for the same individual or monozygotic twins, 0.5 for first-degree relatives, 0.25 for second-degree relatives, 0.125 for third-degree relatives, and 0 for unrelated individuals. Thus, the PI_HAT value of 0.5 between the patient and the father and between the patient and the mother confirms the paternity and maternity, whereas the PI_HAT value of 0 between the father and the mother indicates non-consanguinity between them.

Reference

Purcell S, Neale B, Todd-Brown K, Thomas L, Ferreira MAR, Bender D, et al. PLINK: A tool set for whole-genome association and population-based linkage analyses. Am J Hum Genet. 2007;81(3):559–575.

Supplementary Table S2: Clinical features of our patient and previouly reported patients with *HMGA2* intragenic sequence variants/deletions (Group 1).

Reference	This study	Maharaj et al., 2024	Plachy et al., 2019	Vishnopolska et al., 2021	Hübner et al., 2020	Hübner et al., 2020	Maharaj et al., 2024	Maharaj et al., 2024
Patient	Case 1	Patient 1	Patient 14	Patient 19	Patient 3 (familial case)	Brother (familial case)	Patient 5	Patient 3
Sex	Female	Female	Male	Female	Female	Male	Female	Female
Variant	c.138_141delinsCT, p.(Lys46Asnfs*16)	c.166A>G, p.(Lys56Glu)	c.223C>T, p.(Arg75Trp)	c.223C>T, p.(Arg75Trp)	c.239C>T, p.(Pro80Leu)	c.239C>T, p.(Pro80Leu)	c.49G>T, p.(Gly17*)	c.52C>T, p.(Gln18*)
Variant type	frameshift	missense	missense	missense	missense	missense	nonsense	nonsense
Zygosity	heterozygous	heterozygous	heterozygous	heterozygous	homozygous	homozygous	heterozygous	heterozygous
Inharitance	*de novo*	Inherited from affected mother	NA	NA	paternal/maternal	paternal/maternal	Inherited from affected mother	Inherited from affected mother
**<Netchine-Harbison scoring system features for Silver-Russell syndrome>**								
Birth length and/or weight ≤ –2 SDS	+	+	–	NA	+	+	+	+
Postnatal height ≤ –2 SDS (~ 2 years)	+	+	+	+	+	+	+	+
Relative macrocephaly at birth	+	–	NA	NA	NA	NA	–	–
Prominent forehead (1–3 years)	+	–	NA	NA	+	+	–	+
Body asymmetry	–	–	NA	NA	NA	NA	–	+
Feeding difficulties and/or low BMI	+	+	NA	NA	+	+	+	–
score	5/6	3/6	1/2	1/1	4/4	4/4	3/6	4/6
**<Pregnancy and delivery>**								
Gestational age (weeks)	39	NA	NA	NA	39	38	term	NA
Placental weight - % or SDS	430 g/–0.82	NA	NA	NA	NA	NA	NA	NA
Hypoplastic placenta (<80%)	–	NA	NA	NA	NA	NA	NA	NA
Oligohydramnios	+	NA	NA	NA	NA	NA	NA	NA
**<Growth>**								
Birth length - SDS	46 cm/–1.73	NA	NA/–1.42	NA	36 cm/–6.86	NA	NA	NA
Birth weight - SDS	2140 g/–2.92	NA	NA/–1.94	NA	1400 g/–4.65	1050 g/–5.18	NA	NA
Birth OFC - SDS	31.5 cm/–1.38	NA	NA	NA	NA	NA	NA	NA
Postnatal height - SDS	75.0 cm/–3.17	NA/–3.9 (5.8 years)	NA/–3.06 (5 years)	NA/–2.92 (6 years)	70 cm/–6.73 (3 years)	57 cm/–6.19 (13 months)	NA/–3.6 (5.5 years)	NA/–3.9 (3 years)
Postnatal weight - SDS	7.4 kg/–4.24	NA/–3.8 (5.8 years)	NA	NA	6.2 kg/–7.09 (3 years)	4.1 kg/–6.76 (13 months)	NA/–3.2 (5.5 years)	NA/–2.7 (3 years)
Postnatal OFC - SDS	43.8 cm/–2.0	NA/–4.9 (5.8 years)	NA	NA	NA	NA	NA/–3.9 (5.5 years)	NA/–2.0 (3 years)
**<Craniofacial features not involved in Netchine-Harbison criteria>**								
Triangular face	+	+	NA	NA	NA	NA	–	+
Low set ears or ear anomalies	–	–	NA	NA	+	+	–	–
Cleft palate	–	–	NA	NA	NA	NA	–	–
Micrognathia	+	–	NA	NA	NA	NA	–	–
**<Limb/digital features>**								
Long bone deficiency	–	–	NA	NA	–	–	–	–
Ectrodactyly	–	–	NA	NA	–	–	–	–
Polydactyly	–	–	NA	NA	–	–	–	–
Syndactyly	–	–	NA	NA	–	–	–	–
Clinodactyly	+	–	NA	NA	+	+	–	–
Oateopoikilosis	NA	NA	NA	NA	NA	NA	NA	NA
**<GH therapy>**								
GH therapy	+	–	+	NA	NA	NA	+	+
age at start	3.0 years		NA				5.5 years	9.8 years
GH dose (mg/kg/week)	0.23		8.34 ug/L				NA	0.8→1.0 mg/m^2/day

Supplementary Table S2: Clinical features of our patient and previouly reported patients with *HMGA2* intragenic sequence variants/deletions (Group 1).

Reference	Abi Habib et al., 2018	Maharaj et al., 2024	Maharaj et al., 2024	Abi Habib et al., 2018	Costain et al., 2018	Hübner et al., 2020	Freire et al., 2022	Gorbenko del Blanco et al., 2011
Patient	Patient	Patient 4	Patient 2	Patient	Case 1096	Patient 2	Patient 17	Patient
Sex	Female	Female	Female	Male	Male	Male	NA	Male
Variant	c.193C>T, p.(Gln65*)	c.144del, p.(Arg49Glyfs*117)	c.145del, p.(Arg49Glyfs*117)	c.189del, p.(Ala64Leufs*102)	c.303del, p.(Ser102Hisfs*64)	c.111+1G>T	c.198+1G>C	c.250-29_250-10del
Variant type	nonsense	frameshift	frameshift	frameshift	frameshift	intron	intron	intron
Zygosity	heterozygous	heterozygous	heterozygous	heterozygous	heterozygous	heterozygous	heterozygous	heterozygous
Inharitance	*de novo*	NA	NA	NA	Inherited from affected mother	*de novo*	Inherited from affected mother	NA
**<Netchine-Harbison scoring system features for Silver-Russell syndrome>**								
Birth length and/or weight ≤ –2 SDS	+	+	+	+	+	+	NA	–
Postnatal height ≤ –2 SDS (~ 2 years)	+	+	+	+	+	+	NA	+
Relative macrocephaly at birth	+	–	+	+	–	–	NA	NA
Prominent forehead (1–3 years)	+	+	–	+	–	+	NA	+
Body asymmetry	–	–	–	–	–	–	NA	NA
Feeding difficulties and/or low BMI	+	+	–	+	+	+	NA	NA
score	5/6	4/6	3/6	5/6	3/6	4/6	NA	2/3
**<Pregnancy and delivery>**								
Gestational age (weeks)	39	term	29	37	29+1	39	NA	40
Placental weight - % or SDS	NA	NA	NA	NA	NA	NA	NA	NA
Hypoplastic placenta (<80%)	NA	NA	NA	NA	NA	NA	NA	NA
Oligohydramnios	NA	NA	NA	NA	NA	NA	NA	NA
**<Growth>**								
Birth length - SDS	43.5 cm/–3.9	NA	NA	40 cm/–4.8	33 cm/NA	47 cm/–2.13	NA	50 cm/NA
Birth weight - SDS	2300 g/–2.5	NA/–1.9	NA	1720 g/–3.1	693 g/NA	2360 g/–2.6	NA	3400 g/NA
Birth OFC - SDS	32.5 cm/–2.0	NA	NA	31.0 cm/–2.3	24 cm/NA	31 cm/–3.31	NA	NA
Postnatal height - SDS	66.0 cm/–3.4	NA/–4.1 (7.5 years)	NA/–3.7 (0.5 years)	91.5 cm/–3.0 (4 years)	123 cm/–2.7 (10.7 years)	88 cm/–3.24 (3.8 years)	NA	NA/–4.8 (3 years)
Postnatal weight - SDS	6.85 kg/–3.6	NA/–1.9 (7.5 years)	NA/–3.5 (0.5 years)	11.8 kg/–4.0 (4 years)	25.8 kg/–1.7 (10.7 years)	10.0 kg/–3.93 (3.8 years)	NA	NA
Postnatal OFC - SDS	44.5 cm/–1.2	NA/–1.4 (7.5 years)	NA	NA	49.5 cm/–2.9 (10.7 years)	NA	NA	NA
**<Craniofacial features not involved in Netchine-Harbison criteria>**								
Triangular face	+	–	–	+	+	+	NA	NA
Low set ears or ear anomalies	NA	–	–	NA	NA	–	NA	NA
Cleft palate	NA	–	–	NA	NA	–	NA	NA
Micrognathia	NA	–	–	NA	NA	–	NA	NA
**<Limb/digital features>**								
Long bone deficiency	NA	–	–	NA	–	–	NA	NA
Ectrodactyly	NA	–	–	NA	–	–	NA	NA
Polydactyly	NA	–	–	NA	–	–	NA	NA
Syndactyly	NA	–	–	NA	–	–	NA	NA
Clinodactyly	NA	–	–	NA	–	–	NA	NA
Oateopoikilosis	NA	NA	NA	NA	NA	NA	NA	NA
**<GH therapy>**								
GH therapy	–	+	–	+	–	+	NA	+
age at start		7.5 years		4.2 years		3.8 years		3.0 years
GH dose (mg/kg/week)		NA		0.31		NA		NA

Supplementary Table S2: Clinical features of our patient and previouly reported patients with *HMGA2* intragenic sequence variants/deletions (Group 1).

Reference	De Crescenzo et al., 2015	De Crescenzo et al., 2015	Leszinski et al., 2018	Hübner et al., 2020	Hübner et al., 2020	Buysse et al., 2009	Alyaqoub et al., 2012	Mercadante et al., 2020
Patient	Patient (familial case)	Mother (familial case)	Patient	Patient 1 (familial case)	Father (familial case)	Case 6 #D0811079	Patient	Patient
Sex	Female	Female	Female	Male	Male	Male	Female	Male
Variant	c.283-6_283del	c.283-6_283del	exon 1-2 deletion	exon 1-3 deletion	exon 1-3 deletion	~exon 3 deletion	4.17 Mb deletion including *HMGA2* (exon 4-5 deletion)	exon 5
Variant type	intron	intron	exon deletion	exon deletion	exon deletion	exon deletion	exon deletion	exon deletion
Zygosity	heterozygous	heterozygous	heterozygous	heterozygous	heterozygous	heterozygous	heterozygous	heterozygous
Inharitance	Inherited from affected mother	NA	*de novo*	paternal	NA	Inherited from affected mother	*de novo*	*de novo*
**<Netchine-Harbison scoring system features for Silver-Russell syndrome>**								
Birth length and/or weight ≤ –2 SDS	+	NA	+	+	+	+	+	+
Postnatal height ≤ –2 SDS (~ 2 years)	+	+	+	+	+	+	+	+
Relative macrocephaly at birth	+	–	–	–	+	NA	NA	–
Prominent forehead (1–3 years)	+	+	+	+	+	NA	–	+
Body asymmetry	–	–	–	–	–	–	–	+
Feeding difficulties and/or low BMI	+	+	+	+	–	NA	+	+
score	5/6	3/5	4/6	4/6	4/6	2/3	3/5	5/6
**<Pregnancy and delivery>**								
Gestational age (weeks)	35	NA	39	39+6	42	term	33	39
Placental weight - % or SDS	NA	NA	NA	NA	NA	NA	NA	NA
Hypoplastic placenta (<80%)	NA	NA	NA	NA	NA	NA	NA	NA
Oligohydramnios	NA	NA	NA	NA	NA	NA	+	NA
**<Growth>**								
Birth length - SDS	42.5 cm/–1.3	NA	44 cm/–3.16	46 cm/–2.82	45 cm/–3.5	45 cm/NA	40 cm/NA	47 cm/NA
Birth weight - SDS	1350 g/–2.5	NA	2220 g/–2.6	2365 g/–2.79	2280 g/–3.27	2400 g/NA	1400 g/NA	2350 g/NA
Birth OFC - SDS	31 cm/–1.0	NA	32 cm/–2.0	33 cm/–1.97	34.5 cm/–1.15	NA	NA	31 cm/NA
Postnatal height - SDS	72.5 cm/–3.2 (20 months)	NA/–3.87 (adult)	88.1 cm/–3.5 (4 years)	67 cm/–6.21	160 cm/–2.77 (32 years)	127.5 cm/–2.0 (9 10/12 years)	98 cm/NA (6 years)	81 cm/NA
Postnatal weight - SDS	6.85 kg/–5.7 (20 months)	NA	9.8 kg/–4.4 (4 years)	6.99 kg/–4.57	NA	24.3 kg/–2.0 (9 10/12 years)	11.3 kg/NA (6 years)	7.5 kg/NA
Postnatal OFC - SDS	44.2 cm/–2.2 (20 months)	NA	NA	NA	NA	50 cm/–2.0 (9 10/12 years)	44 cm/NA (6 years)	47.3 cm/NA
**<Craniofacial features not involved in Netchine-Harbison criteria>**								
Triangular face	+	+	+	+	+	–	–	+
Low set ears or ear anomalies	–	–	–	–	–	–	–	+
Cleft palate	–	–	–	–	–	–	–	–
Micrognathia	+	–	–	–	–	–	–	–
**<Limb/digital features>**								
Long bone deficiency	–	–	–	–	–	–	–	–
Ectrodactyly	–	–	–	–	–	–	–	–
Polydactyly	–	–	–	–	–	–	–	–
Syndactyly	+	–	–	–	–	–	–	+
Clinodactyly	+	+	–	–	–	–	–	+
Oateopoikilosis	NA	NA	NA	NA	NA	NA	–	NA
**<GH therapy>**								
GH therapy	NA	NA	NA	NA	–	NA	NA	NA
age at start								
GH dose (mg/kg/week)								

Supplementary Table S3: Clinical features of our patient and previouly reported patients with *HMGA2* -containing deletions (Group 2).

Reference	This study	Buysse et al., 2009	Buysse et al., 2009	Buysse et al., 2009	Buysse et al., 2009	Buysse et al., 2009	Mari et al., 2009
Patient	Case 2	Case 1 #03g1858	Case 2 #D0502619	Case 3 #4818	Case 4 #D0801870	Case 5 #PN07-0349	Patient
Sex	Male	Female	Female	Male	Male	Male	Male
Variant	3.4 Mb deletion including *HMGA2*	6 Mb deletion including *HMGA2*	6 Mb deletion including *HMGA2*	3.44 Mb deletion including *HMGA2*	8.95 Mb deletion including *HMGA2*	3.48 Mb deletion including *HMGA2*	1.83 Mb deletion including *HMGA2*
Variant type	entire gene deletion	entire gene deletion	entire gene deletion	entire gene deletion	entire gene deletion	entire gene deletion	entire gene deletion
Zygosity	heterozygous	heterozygous	heterozygous	heterozygous	heterozygous	heterozygous	heterozygous
Inharitance	*de novo*	*de novo*	*de novo*	*de novo*	*de novo*	*de novo*	*de novo*
**<Netchine-Harbison scoring system features for Silver-Russell syndrom>**							
Birth length and/or weight ≤ –2 SDS	+	+	–	+	+	+	+
Postnatal height ≤ –2 SDS (~ 2 years)	+	+	+	+	+	+	+
Relative macrocephaly at birth	+	–	–	–	–	–	–
Prominent forehead (1–3 years)	–	–	–	–	+	–	+
Body asymmetry	–	–	–	–	–	–	–
Feeding difficulties and/or low BMI	+	NA	NA	NA	+	+	+
score	4/6	2/5	1/5	2/5	4/6	3/6	4/6
**<Pregnancy and delivery>**							
Gestational age (weeks)	39	term	term	term	term	term	36
Placental weight - % or SDS	348 g/–1.65	NA	NA	NA	NA	NA	NA
Hypoplastic placenta (<80%)	+	NA	NA	NA	NA	NA	NA
Oligohydramnios	–	–	NA	NA	–	NA	NA
**<Growth>**							
Birth length - SDS	40.0 cm/–4.19	NA	NA	NA	NA	NA	43 cm/–4.0
Birth weight - SDS	1998 g/–3.38	2060 g/NA	2300g /NA	NA	2550 g/NA	2055 g/NA	1739 g/NA
Birth OFC - SDS	32.0 cm/–0.92	NA	NA	NA	NA	NA	29 cm/–3.66
Postnatal height - SDS	67.7 cm/–5.65	131.5 cm/–6.2 (16 years)	142.3 cm/–3.5 (14 years)	152 cm/–4.0 (18 years)	62 cm/–3.2 (7 months)	74 cm/–2.7 (18 months)	65 cm/–5.3 (18 months)
Postnatal weight - SDS	6.15 kg/-6.91	NA	NA	41 kg/NA (18 years)	5.75 kg/–3.2 (7 months)	6.7 kg/–4.4 (18 months)	5.07 kg/–4.9 (18 months)
Postnatal OFC - SDS	NA	49 cm/–4.4 (16 years)	53.3 cm/–0.66 (14 years)	NA	45.2 cm/0.56 (7 months)	44.1 cm/–2.9 (18 months)	42 cm/–5.6 (18 months)
**<Craniofacial features not involved in Netchine-Harbison criteria>**							
Triangular face	+	–	–	+	–	–	+
Low set ears or ear anomalies	–	–	–	–	–	–	–
Cleft palate	–	–	–	–	–	–	–
Micrognathia	+	+	–	+	–	–	+
**<Limb/digital features>**							
Long bone deficiency	–	–	–	–	–	–	–
Ectrodactyly	–	–	–	–	–	–	–
Polydactyly	–	–	–	–	–	–	–
Syndactyly	–	–	–	–	–	–	–
Clinodactyly	+	–	–	–	–	–	–
Oateopoikilosis	+	+	+	+	–	–	–
**<GH therapy>**							
GH therapy	+	NA	NA	NA	+	NA	+
age at start	3 years				27 months		3 7/12 years
GH dose (mg/kg/week)	0.23→0.30				NA		NA

Supplementary Table S3: Clinical features of our patient and previouly reported patients with *HMGA2* -containing deletions (Group 2).

Reference	Spengler et al., 2009	Lynch et al., 2011	Lynch et al., 2011	Bibb et al., 2012	Bibb et al., 2012	Takenouchi et al., 2012	Nso-Roca et al., 2014
Patient	SR29	Case 1	Case 2	Patient (familial case)	Mother (familial case)	Patient	Patient
Sex	Female	Female	Female	Female	Female	Female	Female
Variant	1.35 Mb deletion including *HMGA2*	10.11 Mb deletion including *HMGA2*	10.12 Mb deletion including *HMGA2*	3.2 Mb deletion including *HMGA2*	3.2 Mb deletion including *HMGA2*	4 Mb deletion including *HMGA2*	8.35 Mb deletion including *HMGA2*
Variant type	entire gene deletion	entire gene deletion	entire gene deletion	entire gene deletion	entire gene deletion	entire gene deletion	entire gene deletion
Zygosity	heterozygous	heterozygous	heterozygous	heterozygous	heterozygous	heterozygous	heterozygous
Inharitance	*de novo*	*de novo*	*de novo*	Inherited from affected mother	NA	*de novo*	*de novo*
**<Netchine-Harbison scoring system features for Silver-Russell syndrom>**							
Birth length and/or weight ≤ –2 SDS	+	–	–	–	+	+	+
Postnatal height ≤ –2 SDS (~ 2 years)	+	+	+	+	+	+	+
Relative macrocephaly at birth	+	–	+	–	–	–	+
Prominent forehead (1–3 years)	+	–	+	–	–	–	+
Body asymmetry	–	–	–	–	–	–	–
Feeding difficulties and/or low BMI	+	+	+	NA	NA	+	+
score	5/6	2/6	4/6	1/5	2/5	3/6	5/6
**<Pregnancy and delivery>**							
Gestational age (weeks)	term	36	37+4	34	36	27+6	38
Placental weight - % or SDS	NA	NA	NA	NA	NA	NA	NA
Hypoplastic placenta (<80%)	NA	NA	NA	NA	NA	NA	NA
Oligohydramnios	NA	NA	NA	NA	NA	NA	NA
**<Growth>**							
Birth length - SDS	46 cm/–2.59	45 cm/NA	NA	43.2 cm/NA	40.6 cm/–2.9	30.5 cm/NA	44 cm/–2.8
Birth weight - SDS	2700 g/–1.83	2050 g/NA	1930 g/NA	1900 g/NA	1900 g/–2.0	527 g/–3.78	1990 g/–2.64
Birth OFC - SDS	NA	32 cm/NA	NA	NA	NA	22.0 cm/NA	33.5 cm/–0.45
Postnatal height - SDS	70.8 cm/–4.5 (1 9/12 years)	101.5 cm/–4.21 (7 11/12 years)	81 cm/–3.1 (3 years)	124 cm/–4.0 (12 years)	93.3 cm/–5.5 (7 years)	76 cm/–3.13 (29 months)	117.3 cm/–1.9 (7 5/12 years)
Postnatal weight - SDS	6.8 kg/–5.4 (1 9/12 years)	14.2 kg/–4.42 (7 11/12 years)	9.9 kg/–3.4 (3 years)	23.3 kg/–3.3 (12 years)	10.9 kg/–5.0 (7 years)	10.5 kg/–0.75 (29 months)	24 kg/–0.7 (7 5/12 years)
Postnatal OFC - SDS	43.7 cm/–3.3 (1 9/12 years)	53.2 cm/+1.13 (7 11/12 years)	NA	45 cm/–5.3 (12 years)	44.5 cm/–5.0 (7 years)	42.5 cm/–3.53 (29 months)	NA
**<Craniofacial features not involved in Netchine-Harbison criteria>**							
Triangular face	+	–	–	–	–	–	+
Low set ears or ear anomalies	+	–	+	+	–	–	–
Cleft palate	–	–	–	–	–	+	–
Micrognathia	–	–	+	+	+	–	–
**<Limb/digital features>**							
Long bone deficiency	–	–	–	–	–	–	–
Ectrodactyly	–	–	–	–	–	–	–
Polydactyly	–	–	–	–	–	–	–
Syndactyly	–	–	–	–	–	–	–
Clinodactyly	+	+	–	+	+	–	–
Oateopoikilosis	–	–	–	–	+	–	–
**<GH therapy>**							
GH therapy	NA	NA	NA	NA	NA	NA	–
age at start							
GH dose (mg/kg/week)							

Supplementary Table S3: Clinical features of our patient and previouly reported patients with *HMGA2* -containing deletions (Group 2).

Reference	Mc Comack et al., 2015	Raymond et al., 2015	Fischetto et al., 2017	Fischetto et al., 2017	Fischetto et al., 2017	Heldt et al., 2018	Heldt et al., 2018
Patient	Patient 1	Patient	Brother 1 (familial case)	Brother 2 (familial case)	Mother (familial case)	Sister (familial case)	Brother (familial case)
Sex	Male	Male	Male	Male	Female	Female	Male
Variant	3.8 Mb deletion including *HMGA2*	t(1;12;14)(q42;q14;q32) deletion including *HMGA2*	1.9 Mb deletion including *HMGA2*	1.9 Mb deletion including *HMGA2*	1.9 Mb deletion including *HMGA2*	1.67 Mb deletion including *HMGA2*	1.67 Mb deletion including *HMGA2*
Variant type	entire gene deletion	entire gene deletion	entire gene deletion	entire gene deletion	entire gene deletion	entire gene deletion	entire gene deletion
Zygosity	heterozygous	heterozygous	heterozygous	heterozygous	heterozygous	heterozygous	heterozygous
Inharitance	NA	*de novo*	Inherited from affected mother	Inherited from affected mother	NA	Inherited from affected mother	Inherited from affected mother
**<Netchine-Harbison scoring system features for Silver-Russell syndrom>**							
Birth length and/or weight ≤ –2 SDS	–	NA	+	+	+	+	+
Postnatal height ≤ –2 SDS (~ 2 years)	+	NA	+	+	+	+	+
Relative macrocephaly at birth	+	NA	–	–	–	–	–
Prominent forehead (1–3 years)	–	NA	–	–	–	+	–
Body asymmetry	–	NA	–	–	–	–	–
Feeding difficulties and/or low BMI	NA	NA	+	+	–	+	+
score	2/5	NA	3/5	3/5	2/6	4/6	3/6
**<Pregnancy and delivery>**							
Gestational age (weeks)	41	32.5	term	term	NA	42	41
Placental weight - % or SDS	NA	NA	NA	NA	NA	NA	NA
Hypoplastic placenta (<80%)	NA	NA	NA	NA	NA	NA	NA
Oligohydramnios	NA	NA	NA	NA	NA	NA	NA
**<Growth>**							
Birth length - SDS	48 cm/NA	39 cm/NA	40 cm/–3.0	40 cm/–3.72	NA	46 cm/–2.81	42 cm/–5.17
Birth weight - SDS	2550 g/NA	1500 g/NA	2050 g/–3.0	1980 g/–2.38	NA	2300 g/–3.12	1950 g/–4.23
Birth OFC - SDS	35 cm/NA	28 cm/NA	NA	30.5 cm/–3.0	NA	NA	NA
Postnatal height - SDS	82.6 cm/–3.5 (3 years)	NA	57 cm/–4.4 (5 months)	81 cm/–4.61 (3 2/12 years)	132 cm/–3.0 (42 years)	144 cm/–3.4 (27 years)	160 cm/–2.15 (29 years)
Postnatal weight - SDS	11 kg/NA (3 years)	NA	5 kg/–3.64 (5 months)	8.5 kg/–5.96 (3 2/12 years)	51 kg/+2.0 (42 years)	31 kg/–4.1 (27 years)	42 kg/–3.5 (29 years)
Postnatal OFC - SDS	49.5 cm/NA (3 years)	NA	NA	47 cm/–2 (3 2/12 years)	NA	49.5 cm/–4.5 (27 years)	52.9 cm/–1.49 (29 years)
**<Craniofacial features not involved in Netchine-Harbison criteria>**							
Triangular face	–	NA	+	+	+	–	–
Low set ears or ear anomalies	–	NA	–	–	–	–	–
Cleft palate	–	NA	–	–	–	–	–
Micrognathia	–	NA	–	+	–	–	–
**<Limb/digital features>**							
Long bone deficiency	–	NA	–	–	–	–	–
Ectrodactyly	–	NA	–	–	–	–	–
Polydactyly	–	NA	–	–	–	–	–
Syndactyly	–	NA	–	–	–	–	–
Clinodactyly	–	NA	–	–	–	–	–
Oateopoikilosis	NA	NA	+	–	+	–	–
**<GH therapy>**							
GH therapy	NA	NA	+	+	–	NA	NA
age at start			2 years	5 years			
GH dose (mg/kg/week)			0.025-0.038 mg/kg/day	NA			

Supplementary Table S3: Clinical features of our patient and previouly reported patients with *HMGA2* -containing deletions (Group 2).

Reference	Heldt et al., 2018	Cottrell et al., 2020
Patient	Mother (familial case)	Patient 3
Sex	Female	Male
Variant	1.67 Mb deletion including *HMGA2*	3.38 Mb deletion including *HMGA2*
Variant type	entire gene deletion	entire gene deletion
Zygosity	heterozygous	heterozygous
Inharitance	NA	NA
**<Netchine-Harbison scoring system features for Silver-Russell syndrom>**		
Birth length and/or weight ≤ –2 SDS	NA	–
Postnatal height ≤ –2 SDS (~ 2 years)	+	+
Relative macrocephaly at birth	–	–
Prominent forehead (1–3 years)	–	–
Body asymmetry	–	–
Feeding difficulties and/or low BMI	+	+
score	2/5	2/6
**<Pregnancy and delivery>**		
Gestational age (weeks)	NA	NA
Placental weight - % or SDS	NA	NA
Hypoplastic placenta (<80%)	NA	NA
Oligohydramnios	NA	NA
**<Growth>**		
Birth length - SDS	NA	NA
Birth weight - SDS	NA	NA
Birth OFC - SDS	NA	NA
Postnatal height - SDS	148 cm/–2.59 (52 years)	NA/–5.1 (11.3 years)
Postnatal weight - SDS	42 kg/–1.8 (52 years)	NA/–1.9 (11.3 years)
Postnatal OFC - SDS	49.2 cm/–4.86 (52 years)	NA
**<Craniofacial features not involved in Netchine-Harbison criteria>**		
Triangular face	–	+
Low set ears or ear anomalies	–	NA
Cleft palate	–	NA
Micrognathia	–	NA
**<Limb/digital features>**		
Long bone deficiency	–	NA
Ectrodactyly	–	NA
Polydactyly	–	NA
Syndactyly	–	NA
Clinodactyly	–	NA
Oateopoikilosis	–	NA
**<GH therapy>**		
GH therapy	NA	NA
age at start		
GH dose (mg/kg/week)		

Supplementary Table S4: Clinical features of previouly reported patients with *PLAG1* intragenic sequence variants (Group 3).

Reference	Inoue et al., 2020	Dong et al., 2023	Dong et al., 2023	Tse et al., 2023	Tse et al., 2023	Abi Habib et al., 2018	Abi Habib et al., 2018
Patient	Patient 4	Patient (familial case)	Father (familial case)	Patient (familial case)	Mother (familial case)	Patient I-2 (familial case)	Patient II-1 (familial case)
Sex	Female	Female	Male	Female	Female	Female	Female
Variant	c.589C>T, p.(Arg197*)	c.131del, p.(Asn44Thrfs*6)	c.131del, p.(Asn44Thrfs*6)	c.402del, p.(Gly135Aspfs*94)	c.402del, p.(Gly135Aspfs*94)	c.439del, p.(Ser147Valfs*82)	c.439del, p.(Ser147Valfs*82)
Variant type	nonsense	frameshift	frameshift	frameshift	frameshift	frameshift	frameshift
Zygosity	heterozygous	heterozygous	heterozygous	heterozygous	heterozygous	heterozygous	heterozygous
Inharitance	Inherited from affected mother	Inherited from affected father	NA	Inherited from affected mother	NA	NA	Inherited from affected mother
**<Netchine-Harbison scoring system features for Silver-Russell syndrome>**							
Birth length and/or weight ≤ –2 SDS	+	+	+	+	+	+	+
Postnatal height ≤ –2 SDS (~ 2 years)	+	+	+	NA	NA	+	+
Relative macrocephaly at birth	+	+	NA	NA	NA	NA	–
Prominent forehead (1–3 years)	+	+	NA	NA	NA	+	–
Body asymmetry	–	–	–	NA	NA	–	–
Feeding difficulties and/or low BMI	+	–	+	+	+	NA	+
score	5/6	4/6	3/4	2/2	2/2	3/4	3/6
**<Pregnancy and delivery>**							
Gestational age (weeks)	38+5	39+5	40+1	37+3	40	Term	39+6
Placental weight - % or SDS	NA	NA	NA	NA	NA	NA	NA
Hypoplastic placenta (<80%)	NA	NA	NA	NA	NA	NA	NA
Oligohydramnios	NA	NA	NA	NA	NA	NA	NA
**<Growth>**							
Birth length - SDS	42.0 cm/–3.12	44 cm/–3.05	NA	NA	NA	NA	44.5 cm/–2.3
Birth weight - SDS	1804 g/–3.28	2700 g/–1.48	2450 g/–2.05	2020 g/NA	2040 g/NA	NA	2340 g/–2.1
Birth OFC - SDS	32.0 cm/–0.86	33.6 cm/–0.81	NA	NA	NA	NA	31 cm/–3.84
Postnatal height - SDS	77.6 cm/–3.42	79 cm/–2.32 (25 months)	159 cm/–2.14 (28 years)	NA	155 cm/NA (25 years)	NA	79 cm/–2.0
Postnatal weight - SDS	NA	8.9 kg/–2.37 (25 months)	NA	NA	NA	NA	8.52 kg/–3.7
Postnatal OFC - SDS	NA	45.5 cm/–1.3 (25 months)	NA	NA	NA	NA	43.9 cm/–2.6
**<Craniofacial features not involved in Netchine-Harbison criteria>**							
Triangular face	+	+	–	NA	NA	+	+
Low set ears or ear anomalies	–	–	–	NA	NA	NA	NA
Cleft palate	–	–	–	NA	NA	NA	NA
Micrognathia	–	–	–	NA	NA	NA	NA
**<Limb/digital features>**							
Long bone deficiency	–	–	–	NA	NA	NA	NA
Ectrodactyly	–	–	–	NA	NA	NA	NA
Polydactyly	–	–	–	NA	NA	NA	NA
Syndactyly	–	–	–	NA	NA	NA	NA
Clinodactyly	+	–	–	NA	NA	NA	NA
Oateopoikilosis	NA	NA	NA	NA	NA	NA	NA
**<GH therapy>**							
GH therapy	+	– (34 months)	NA	NA	NA	–	+
age at start	3.0 years						2.5 years
GH dose (mg/kg/week)	NA						0.26

Supplementary Table S4: Clinical features of previouly reported patients with *PLAG1* intragenic sequence variants (Group 3).

Reference	Abi Habib et al., 2018	Vado et al., 2020	Meyer et al., 2021	Abi Habib et al., 2018
Patient	Patient II-2 (familial case)	Patient	Patient 1	Sporadic case
Sex	Female	Female	Female	Female
Variant	c.439del, p.(Ser147Valfs*82)	c.551del, p.(Lys184Serfs*45)	c.599dup, p.(Arg201Profs*52)	c.1363del, p.(Gln455Serfs*16)
Variant type	frameshift	frameshift	frameshift	frameshift
Zygosity	heterozygous	heterozygous	heterozygous	heterozygous
Inharitance	Inherited from affected mother	Inherited from affected mother	*de novo*	*de novo*
**<Netchine-Harbison scoring system features for Silver-Russell syndrome>**				
Birth length and/or weight ≤ –2 SDS	+	+	+	+
Postnatal height ≤ –2 SDS (~ 2 years)	+	NA	NA	+
Relative macrocephaly at birth	–	–	+	+
Prominent forehead (1–3 years)	+	NA	+	+
Body asymmetry	–	–	–	–
Feeding difficulties and/or low BMI	+	+	+	+
score	4/6	2/4	4/5	5/6
**<Pregnancy and delivery>**				
Gestational age (weeks)	39	38	36	31+6
Placental weight - % or SDS	NA	small placenta	NA	NA
Hypoplastic placenta (<80%)	NA	NA	NA	NA
Oligohydramnios	NA	NA	NA	NA
**<Growth>**				
Birth length - SDS	47 cm/–2.0	41 cm/–4.03	39 cm/–3.12	37.5 cm/–2.78
Birth weight - SDS	2557 g/–1.9	1600 g/–4.24	1370 g/–3.12	1050 g/–3.0
Birth OFC - SDS	31 cm/–3.45	27 cm/–5.61	30.5 cm/–2.2	27.4 cm/–1.5
Postnatal height - SDS	75 cm/–2.8	NA	71 cm/–3.6 (19 months)	79 cm/–2.0
Postnatal weight - SDS	6.6 kg/–6.7	NA	7.4 kg/–3.0 (19 months)	7.8 kg/–4.4
Postnatal OFC - SDS	43.3 cm/–2.5	NA	45 cm/–2.07 (19 months)	44.7 cm/–2.0
**<Craniofacial features not involved in Netchine-Harbison criteria>**				
Triangular face	+	+	+	+
Low set ears or ear anomalies	NA	–	–	NA
Cleft palate	NA	–	–	NA
Micrognathia	NA	–	+	NA
**<Limb/digital features>**				
Long bone deficiency	NA	–	–	NA
Ectrodactyly	NA	–	–	NA
Polydactyly	NA	–	–	NA
Syndactyly	NA	–	–	NA
Clinodactyly	NA	–	–	NA
Oateopoikilosis	NA	NA	NA	NA
**<GH therapy>**				
GH therapy	+	NA	NA	+
age at start	2.0 years			6.0 years
GH dose (mg/kg/week)	0.27			0.24

Supplementary Table S5: Clinical features of previouly reported patients with *PLAG1* -containing deletions (Group 4).

Reference	Schinzel et al., 1994	Brereton et al., 2021	Brereton et al., 2021	Fernandez-Fructuoso et al., 2021	Baba et al., 2022
Patient	Patient	Patient 1 (siblings)	Patient 2 (siblings)	Patient	Case 2
Sex	Female	Male	Male	Female	Male
Variant	46,XX,del[8](q11q12)	46,XY.ish rec(8)(pter->q12.1::q12.1->qter)	45,XY,der(8)t(8;21)(p23.1; q11.2)	2.1 Mb deletion including *PLAG1*	77 kb deletion including *PLAG1*
Variant type	deletion	entire gene deletion	entire gene deletion	entire gene deletion	entire gene deletion
Zygosity	heterozygous	heterozygous	heterozygous	heterozygous	heterozygous
Inharitance	NA	(Maternal)	(Maternal)	*de novo*	*de novo*
**<Netchine-Harbison scoring system features for Silver-Russell syndrome>**					
Birth length and/or weight ≤ –2 SDS	+	–	+	+	–
Postnatal height ≤ –2 SDS (~ 2 years)	+	NA	–	+	–
Relative macrocephaly at birth	–	+	+	+	–
Prominent forehead (1–3 years)	+	+	+	–	+
Body asymmetry	–	–	–	–	–
Feeding difficulties and/or low BMI	NA	+	+	+	+
score	3/5	3/5	4/6	4/6	2/6
**<Pregnancy and delivery>**					
Gestational age (weeks)	36	38+2	36+6	32	30
Placental weight - % or SDS	NA	NA	NA	NA	NA
Hypoplastic placenta (<80%)	NA	NA	NA	NA	NA
Oligohydramnios	NA	NA	NA	NA	NA
**<Growth>**					
Birth length - SDS	35.5 cm/NA	45 cm/–1.58	41 cm/–2.98	34.5 cm/–4.76	37 cm/–1.39
Birth weight - SDS	1100 g/NA	2285 g/–1.5	1700 g/–2.43	950 g/–3.50	990 g/–1.79
Birth OFC - SDS	26.0 cm/NA	34 cm/0.51	31 cm/–1.13	26.5 cm/–2.30	27 cm/–0.23
Postnatal height - SDS	120 cm/NA (9 9/12 years)	103 cm/–1.26 (5 years)	82.9 cm/–1.65 (27 months)	76 cm/–3.89 (26 months)	80 cm/–1.45 (19 months)
Postnatal weight - SDS	14.8 kg/NA (9 9/12 years)	13.9 kg/–2.5 (5 years)	9 kg/–3.64 (27 months)	7.16 kg/–3.97 (26 months)	7.1 kg/–3.87 (19 months)
Postnatal OFC - SDS	46.8 cm/NA (9 9/12 years)	NA	NA	46 cm/–1.99 (26 months)	44 cm/–4.06 (19 months)
**<Craniofacial features not involved in Netchine-Harbison criteria>**					
Triangular face	+	+	+	+	+
Low set ears or ear anomalies	+	–	–	+	+
Cleft palate	–	–	–	–	–
Micrognathia	–	–	–	+	–
**<Limb/digital features>**					
Long bone deficiency	–	–	–	–	–
Ectrodactyly	–	–	–	–	–
Polydactyly	–	–	–	–	–
Syndactyly	–	–	–	–	–
Clinodactyly	+	+	+	–	–
Oateopoikilosis	NA	NA	NA	NA	NA
**<GH therapy>**					
GH therapy	NA	NA	NA	NA	NA
age at start					
GH dose (mg/kg/week)					

Supplementary References

HMGA2 intragenic sequence variants/deletions (Group 1)

1. Buysse K, Reardon W, Mehta L, Costa T, Fagerstrom C, Kingsbury DJ, et al. The 12q14 microdeletion syndrome: additional patients and further evidence that HMGA2 is an important genetic determinant for human height. Eur J Med Genet. 2009;52(2-3):101-107.
2. Gorbenko del Blanco D, de Graaff LC, Posthouwer D, Visser TJ, Hokken-Koelega AC. Isolated GH deficiency: mutation screening and copy number analysis of HMGA2 and CDK6 genes. Eur J Endocrinol. 2011;165(4):537-544.
3. Alyaqoub F, Pyatt RE, Bailes A, Brock A, Deeg C, McKinney A, et al. 12q14 microdeletion associated with HMGA2 gene disruption and growth restriction. Am J Med Genet A. 2012;158A(11):2925-2930.
4. De Crescenzo A, Citro V, Freschi A, Sparago A, Palumbo O, Cubellis MV, et al. A splicing mutation of the HMGA2 gene is associated with Silver-Russell syndrome phenotype. J Hum Genet. 2015;60(6):287-293.
5. Abi Habib W, Brioude F, Edouard T, Bennett JT, Lienhardt-Roussie A, Tixier F, et al. Genetic disruption of the oncogenic HMGA2-PLAG1-IGF2 pathway causes fetal growth restriction. Genet Med. 2018;20(2):250-258.
6. Costain G, Jobling R, Walker S, Reuter MS, Snell M, Bowdin S, et al. Periodic reanalysis of whole-genome sequencing data enhances the diagnostic advantage over standard clinical genetic testing. Eur J Hum Genet. 2018;26(5):740-744.
7. Leszinski GS, Warncke K, Hoefele J, Wagner M. A case report and review of the literature indicate that HMGA2 should be added as a disease gene for Silver-Russell syndrome. Gene. 2018;663:110-114.
8. Plachy L, Strakova V, Elblova L, Obermannova B, Kolouskova S, Snajderova M, et al. High prevalence of growth plate gene variants in children with familial short stature treated with GH. J Clin Endocrinol Metab. 2019;104(10):4273-4281.
9. Hübner CT, Meyer R, Kenawy A, Ambrozaityte L, Matuleviciene A, Kraft F, et al. HMGA2 variants in Silver-Russell syndrome: homozygous and heterozygous occurrence.

J Clin Endocrinol Metab. 2020;105(7):dgaa273.
10. Mercadante F, Busè M, Salzano E, Fragapane T, Palazzo D, Malacarne M, et al. 12q14.3 microdeletion involving HMGA2 gene cause a Silver-Russell syndrome-like phenotype: a case report and review of the literature. Ital J Pediatr. 2020;46(1):108.
11. Vishnopolska SA, Mercogliano MF, Camilletti MA, Mortensen AH, Braslavsky D, Keselman A, et al. Comprehensive identification of pathogenic gene variants in patients with neuroendocrine disorders. J Clin Endocrinol Metab. 2021;106(7):1956-1976.
12. Freire BL, Homma TK, Lerario AM, Seo GH, Han H, de Assis Funari MF, et al. High frequency of genetic/epigenetic disorders in short stature children born with very low birth weight. Am J Med Genet A. 2022;188(9):2599-2604.
13. Maharaj AV, Cottrell E, Thanasupawat T, Joustra SD, Triggs-Raine B, Fujimoto M, et al. Characterization of HMGA2 variants expands the spectrum of Silver-Russell syndrome. JCI Insight. 2024;9(6):e169425.

***HMGA2*-containing deletions (Group 2)**

14. Buysse K, Reardon W, Mehta L, Costa T, Fagerstrom C, Kingsbury DJ, et al. The 12q14 microdeletion syndrome: additional patients and further evidence that HMGA2 is an important genetic determinant for human height. Eur J Med Genet. 2009;52(2-3):101-107.
15. Mari F, Hermanns P, Giovannucci-Uzielli ML, Galluzzi F, Scott D, Lee B, et al. Refinement of the 12q14 microdeletion syndrome: primordial dwarfism and developmental delay with or without osteopoikilosis. Eur J Hum Genet. 2009;17(9):1141-1147.
16. Spengler S, Schönherr N, Binder G, Wollmann HA, Fricke-Otto S, Mühlenberg R, et al. Submicroscopic chromosomal imbalances in idiopathic Silver-Russell syndrome (SRS): the SRS phenotype overlaps with the 12q14 microdeletion syndrome. J Med Genet. 2010;47(5):356-360.
17. Lynch SA, Foulds N, Thuresson AC, Collins AL, Annerén G, Hedberg BO, et al. The 12q14 microdeletion syndrome: six new cases confirming the role of HMGA2 in growth. Eur J Hum Genet. 2011;19(5):534-539.

18. Bibb AL, Rosenfeld JA, Weaver DD. Report of a mother and daughter with the 12q14 microdeletion syndrome. Am J Med Genet A. 2012;158A(2):417-422.
19. Takenouchi T, Enomoto K, Nishida T, Torii C, Okazaki T, Takahashi T, et al. 12q14 microdeletion syndrome and short stature with or without relative macrocephaly. Am J Med Genet A. 2012;158A(10):2542-2544.
20. Nso-Roca AP, Marco FC, Ricote JM, Ruiz MJ. Endocrinological anomalies in a patient with 12q14 microdeletion syndrome. Completing phenotype of this exceptional short stature condition. J Pediatr Endocrinol Metab. 2014;27(5-6):539-543.
21. Mc Cormack A, Sharpe C, Gregersen N, Smith W, Hayes I, George AM, et al. 12q14 Microdeletions: Additional Case Series with Confirmation of a Macrocephaly Region. Case Rep Genet. 2015;2015:192071.
22. Raymond L, Francou B, Petit F, Tosca L, Briand-Suleau A, Metay C, et al. Complex translocation t(1;12;14)(q42;q14;q32) and HMGA2 deletion in a fetus presenting growth delay and bilateral cataracts. Eur J Med Genet. 2015;58(11):591-596.
23. Fischetto R, Palumbo O, Ortolani F, Palumbo P, Leone MP, Causio FA, et al. Clinical and molecular characterization of a second family with the 12q14 microdeletion syndrome and review of the literature. Am J Med Genet A. 2017;173(7):1922-1930.
24. Heldt F, Wallaschek H, Ripperger T, Morlot S, Illig T, Eggermann T, et al. 12q14 microdeletion syndrome: A family with short stature and Silver-Russell syndrome (SRS)-like phenotype and review of the literature. Eur J Med Genet. 2018;61(8):421-427.
25. Cottrell E, Cabrera CP, Ishida M, Chatterjee S, Greening J, Wright N, et al. Rare CNVs provide novel insights into the molecular basis of GH and IGF-1 insensitivity. Eur J Endocrinol. 2020;183(6):581-595.

***PLAG1* intragenic sequence variants (Group 3)**

26. Abi Habib W, Brioude F, Edouard T, Bennett JT, Lienhardt-Roussie A, Tixier F, et al. Genetic disruption of the oncogenic HMGA2-PLAG1-IGF2 pathway causes fetal growth restriction. Genet Med. 2018;20(2):250-258.
27. Inoue T, Nakamura A, Iwahashi-Odano M, Tanase-Nakao K, Matsubara K, Nishioka J, et

al. Contribution of gene mutations to Silver-Russell syndrome phenotype: multigene sequencing analysis in 92 etiology-unknown patients. Clin Epigenetics. 2020;12(1):86.
28. Vado Y, Pereda A, Llano-Rivas I, Gorria-Redondo N, Díez I, Perez de Nanclares G. Novel Variant in PLAG1 in a Familial Case with Silver-Russell Syndrome Suspicion. Genes (Basel). 2020;11(12):1461.
29. Meyer R, Begemann M, Hübner CT, Dey D, Kuechler A, Elgizouli M, et al. One test for all: whole exome sequencing significantly improves the diagnostic yield in growth retarded patients referred for molecular testing for Silver-Russell syndrome. Orphanet J Rare Dis. 2021;16(1):42.
30. Dong P, Zhang N, Zhang Y, Liu CX, Li CL. Clinical characterization of PLAG1- related Silver-Russell syndrome：A clinical report. Eur J Med Genet. 2023;66(10):104837.
31. Tse WT, Bass C, Gurney L, Kinning E. Maternally inherited autosomal dominant PLAG-1 related Silver Russell syndrome in a fetus with intra-uterine growth restriction. Prenat Diagn. 2023;43(6):724-726.

***PLAG1*-containing deletions (Group 4)**

32. Schinzel AA, Robinson WP, Binkert F, Fanconi A. An interstitial deletion of proximal 8q (q11-q13) in a girl with Silver-Russell syndrome-like features. Clin Dysmorphol. 1994;3(1):63-69.
33. Brereton RE, Nickerson SL, Woodward KJ, Edwards T, Sivamoorthy S, Ramos Vasques Walters F, et al. Further heterogeneity in Silver-Russell syndrome: PLAG1 deletion in association with a complex chromosomal rearrangement. Am J Med Genet A. 2021;185(10):3136-3145.
34. Fernández-Fructuoso JR, De la Torre-Sandoval C, Harbison MD, Chantot-Bastaraud S, Temple K, Lloreda-Garcia JM, et al. Silver Russell syndrome in a preterm girl with 8q12.1 deletion encompassing PLAG1. Clin Dysmorphol. 2021;30(4):194-196.
35. Baba N, Lengyel A, Pinti E, Yapici E, Schreyer I, Liehr T, et al. Microdeletions in 1q21 and 8q12.1 depict two additional molecular subgroups of Silver-Russell syndrome like phenotypes. Mol Cytogenet. 2022;15(1):19.
